# Novel Polyimide/Copper-Nickel Ferrite Composites with Tunable Magnetic and Dielectric Properties

**DOI:** 10.3390/polym13101646

**Published:** 2021-05-19

**Authors:** Corneliu Hamciuc, Mihai Asandulesa, Elena Hamciuc, Tiberiu Roman, Marius Andrei Olariu, Aurel Pui

**Affiliations:** 1 “Petru Poni” Institute of Macromolecular Chemistry, 41A Aleea Gr. Ghica Voda, 700487 Iasi, Romania; chamciuc@icmpp.ro (C.H.); asandulesa.mihai@icmpp.ro (M.A.); ehamciuc@icmpp.ro (E.H.); 2 Faculty of Chemistry, “Al. I. Cuza” University of Iasi, 11 Bd. Carol I, 700506 Iasi, Romania; tiberiu.roman@chem.uaic.ro; 3 Integrated Centre of Environmental Science Studies in the North-Eastern Region—CERNESIM, “Al. I. Cuza” University of Iasi, 11 Bd. Carol I, 700506 Iasi, Romania; 4 Prosupport Consulting SRL, 29 Peter Culianu Street, Valea Lupului, 707410 Iasi, Romania; marius.olariu@prosupport-consulting.ro; 5 Faculty of Electrical Engineering, “Gh. Asachi” Technical University, B-Dul D. Mangeron 67, 700050 Iasi, Romania

**Keywords:** composites, ferrimagnetic enhancement behavior, structural analysis, dielectric properties

## Abstract

Heat-resistant magnetic polymer composites were prepared by incorporating cerium-doped copper-nickel ferrite particles, having the general formula Ni_1-x_Cu_x_Fe_1.92_Ce_0.08_O_4_ (x: 0.0, 0.3, 0.6, 1.0), into a polyimide matrix. The effects of particle type and concentration on the thermal, magnetic, and electrical properties of the resulting composites were investigated. The samples were characterized by FTIR, scanning electron microscopy, X-ray diffractometry, thermogravimetric analysis, differential scanning calorimetry, vibrating sample magnetometer, and broadband dielectric spectroscopy. The composites exhibited high thermal stability, having initial decomposition temperatures between 495 and 509 °C. Saturation magnetization (*M_s_*), magnetic remanence (*M_r_*), and coercivity (*H_c_*) were found in range of 2.37–10.90 emu g^−1^, 0.45–2.84 emu g^−1^, and 32–244 Oe, respectively. The study of dielectric properties revealed dielectric constant values of 3.0–4.3 and low dielectric losses of 0.016–0.197 at room temperature and a frequency of 1 Hz.

## 1. Introduction

With exceptional properties resulting from a unique combination of organic and inorganic components, polymer nanocomposites are of great importance in current material chemistry research. In this regard, the polymeric phase confers flexibility and good processability, while the inorganic phase gives them thermal, optical, electrical, or magnetic characteristics [1]. Magnetic polymer nanocomposites are a particularly well-studied subgroup, since they can easily be prepared by incorporating magnetic inorganic nanoparticles into a polymer matrix, and have been extensively explored in a wide variety of high-performance applications, including in vivo imaging, photocatalysis, and heavy metal absorption [2,3,4,5].

Cobalt ferrite (CoFe_2_O_4_) is one of the most important spinel ferrites, having a high Curie temperature, relatively large magnetic hysteresis, moderate magnetic saturation, high coercivity, and high thermal stability. CoFe_2_O_4_ found its application in many fields including energy conversion and storage, magnetic recording and high-density digital recording disks, gas sensing, catalysis, biotechnology, magnetic bulk cores, and microwave absorbers [6,7]. It has also found use in biomedical applications such as magnetic thermo-drug delivery, hyperthermia, biosensors, and magnetic resonance imaging [8,9,10,11]. Besides Co^2+^, various other metallic cations such as Ni^2+^, Cu^2+^, Zn^2+^, Mg^2+^, Mn^2+^, have been partially or even totally introduced into the ferritic structure (M^2+^Fe_2_O_4_), resulting in nanoparticles with specific morphology and properties [12,13,14,15,16,17]. For example, it was found that for Co_1-x_Ni_x_Fe_2_O_4_ nanofibers, saturation magnetization, and coercivity decreased with increasing nickel concentration due to the smaller magnetic moment of Ni^2+^ ions compared to Co^2+^ ions [12]. At the same time, saturation magnetization decreased at Co_1-x_Cu_x_Fe_2_O_4_ nanoparticles by increasing Cu^2+^ content, due to the smaller magnetic moment of Cu^2+^ ions in comparison with Co^2+^ ions [13]. Several studies reported that cerium insertion into the ferrite structure leads to an enhancement of its electrical and magnetic properties, which makes these nanoparticles more suitable for use in microwave adsorption and photocatalytic applications [18,19]. Moreover, calcination at 600 °C with only a small quantity of cerium slightly increases the ferrites’ magnetic properties, as our previous work demonstrates for CuFe_1.92_Ce_0.08_O_4_ [20], NiFe_1.92_Ce_0.08_O_4_, Ni_0.7_Cu_0.3_Fe_1.92_Ce_0.08_O_4_, and Ni_0.4_Cu_0.6_Fe_1.92_Ce_0.08_O_4_ complex systems [21].

Aromatic polyimides represent the most important class of thermostable heterocyclic polymers due to their outstanding properties (e.g., high thermo-oxidative resistance, high glass transition temperatures, very good mechanical, and electrical properties) and their use in various technical applications (e.g., aerospace and microelectronics) [22,23,24,25]. Due to their aromatic rigid structure, polyimides are often insoluble and infusible and thus can be processed with difficulty. The incorporation of flexible linkages into their main chains, such as aromatic ether groups, can substantially improve the processability of these polymers. The introduction of magnetic inorganic particles into a polyimide matrix can produce materials with improved properties exhibiting high thermal stability, excellent mechanical behavior, magnetic properties, and flexibility [26,27,28,29,30,31,32,33,34]. Flexible magnetic polyimide films are important for the fabrication of flexible electronics, devices which have been developed rapidly in the last years.

In this study, the preparation and characterization of novel heat-resistant magnetic polyimide composites based on cerium-doped copper-nickel ferrite particles are reported. An imide-copolymer having pendant carboxylic groups and ether linkages was used as the polymer matrix. The effect of ferrite particles on the thermal, magnetic, and electrical properties of the resulting composites was also investigated. We show that the content and type of ferrite particles allow the fine tuning of magnetic and dielectric characteristics of these composites. Finally, we discuss the sub-glass and glass-rubber transitions, the conductivity as well as the changes produced in the relaxation processes.

## 2. Experimental

### 2.1. Materials and Methods

#### 2.1.1. Materials

4,4′-Oxydiphthalic anhydride, 2,2-bis [4-(4-aminophenoxy)phenyl]propane, 3,5-diaminobenzoic acid, and *N*-methyl-2-pyrrolidone (NMP) were purchased from Aldrich and used as received. Cerium-doped copper-nickel ferrite particles were prepared as previously described [20,21]. Table 1 illustrates the chemical structure and main magnetic characteristics of the particles: saturation magnetization (*M_s_*), remanence magnetization (*M_r_*), and coercivity (*H_c_*).

#### 2.1.2. Preparation of Polyimide Composites

**Figure 1:**
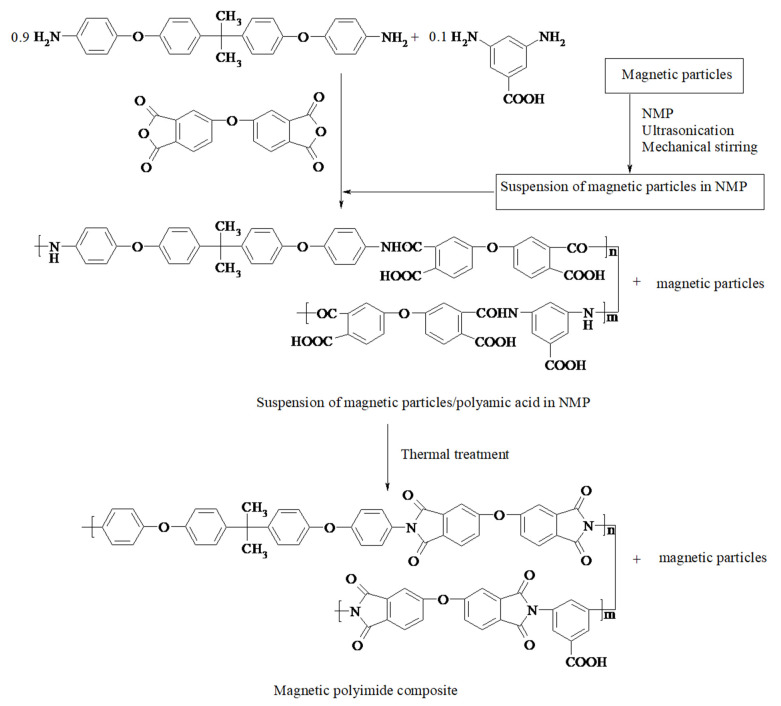
Preparation of magnetic polyimide composites.

Magnetic polyimide composites were prepared by incorporating magnetic particles into a poly(amic acid) solution, followed by film casting and thermal imidization, in accordance with our previous work (Figure 1) [35]. Magnetic particles and NMP (11 mL) were introduced into a flask and the resulting mixture was sonicated for 2 h, after which it was mechanically stirred for 6 h until a suspension was obtained. Afterwards, 2,2-bis[4-(4-aminophenoxy)phenyl]propane (0.738 g, 0.0018 mol) and 3,5-diaminobenzoic acid (0.0104 g, 0.0002 mol) were added, and the mixture was stirred under nitrogen to complete dissolution. Furthermore, 4,4′-oxydiphthalic anhydride (0.620 g, 0.002 mol) was introduced and stirring was continued at room temperature for 12 h. Entrapped air bubbles were removed under vacuum. The suspension was cast onto glass plates and heated at 50, 100, 150, 200 °C, for 1 h each, and at 250 °C for 2 h. The inorganic content of the composites was controlled by the portion ratio between ferrite particles and poly(amic acid). The concentration of particles was calculated considering the appropriate amount of poly(amic acid) corresponding to final imide structure of the polymer matrix. Four polyimide composites PI-A-10, PI-A-20, PI-A-30, and PI-A-40 containing ferrite particles of type A in concentration of 10, 20, 30, and 40 wt%, respectively, and three polyimide composites PI-B-30, PI-C-30, and PI-D-30 with 30 wt% ferrite particles of type B, C, and D, respectively, were prepared (Table 2). A reference polyimide film without magnetic particles PI-0 was prepared as well.

#### 2.1.3. Measurements

FTIR spectra were recorded on a Bruker Vertex 70 Spectrometer at frequencies ranging from 400 to 4000 cm^−1^.

Microscopic investigations were performed on an environmental scanning electron microscope type Quanta 200 operating at 10 kV with secondary and backscattering electrons in low vacuum mode. The Quanta 200 microscope is equipped with an energy dispersive X-ray (EDX) system for qualitative and quantitative analysis, as well as elemental mapping.

X-ray diffraction measurements (XRD) were carried out on a XpertPanalytical Pro MPD diffractometer equipped with a copper source radiation (Cu-Kα1 radiation, λ = 1.5406 Å) in 10–80° over 2θ range at room temperature.

Thermogravimetric (TG) curves and thermogravimetric derivative (DTG) curves were recorded with a Mettler Toledo TGA-SDTA851^e^ equipment, in nitrogen atmosphere, under dynamic conditions with a flow rate of 20 mL min^−1^ and a heating rate of 10 °C min^−1^, in the temperature range of 25–900 °C and with a sample mass between 2.6 and 4.7 mg.

Differential scanning calorimetry (DSC) curves were obtained using a Mettler Toledo DSC1 type device in an inert atmosphere, with a heating rate of 10 °C min^−1^ and nitrogen purge at 100 mL min^−1^. Scanning was performed in the temperature range of 25–350 °C. The mass of samples encapsulated in aluminum pans having pierced lids to allow the evaporation of the volatile components was between 2.4 and 4.7 mg. The evaluation of TG and DSC curves was performed with STAR^e^ software, version 9, of Mettler-Toledo.

Room temperature magnetic properties were investigated in a ±10 kOe range using a vibrating sample magnetometer (VSM) (AGM & VSM Magnetometer, MicroMag 3900 series from former Princeton Measurement Co., currently Lake Shore Cryotronics, Inc., Westerville, OH, USA).

Broadband dielectric spectroscopy measurements were carried out on a dielectric spectrometer (Novocontrol Technologies, GmbH@Co., Montabaur, Germany) in a broad range of frequency (from 1 to 10^6^ Hz) and temperature (from −150 to 250 °C). The alternating electrical field was applied with an Alpha-A High Performance Frequency Analyzer device and the temperature was controlled with 0.1 °C stability and high reproducibility using a Novocontrol Quatro Cryo system device. Composite samples were sandwiched between two gold-plated flat electrodes, and the measurements were performed in dry nitrogen.

## 3. Results and Discussions

### 3.1. Structural and Morphological Characterization

The chemical structure of the samples was confirmed by FTIR spectroscopy. Characteristic absorption bands of imide rings were observed in all spectra at around 1770 cm^−1^ (imide carbonyl asymmetric stretching), 1715 cm^−1^ (imide carbonyl symmetric stretching and C=O stretching of carboxylic pendant groups), 1370 cm^−1^ (C-N stretching), and 742 cm^−1^ (C=O bending). All the spectra showed absorption bands at 2970 and 2865 cm^−1^ (C-H of isopropylidene groups), 3060 cm^−1^ (C-H aromatic), and 1603 and 1501 cm^−1^ (-C=C- aromatic). The characteristic band for amide carbonyl at 1650 cm^−1^ was not observed in the spectra, suggesting that the imidization reaction took place. The FTIR spectra of composites were similar to that of reference polyimide PI-0, indicating that there is no chemical bonding between particles and polymer, as well as no degradation of the polymer matrix (Figure S1 from the Electronic Supplementary Data, ESD).

**Figure 2:**
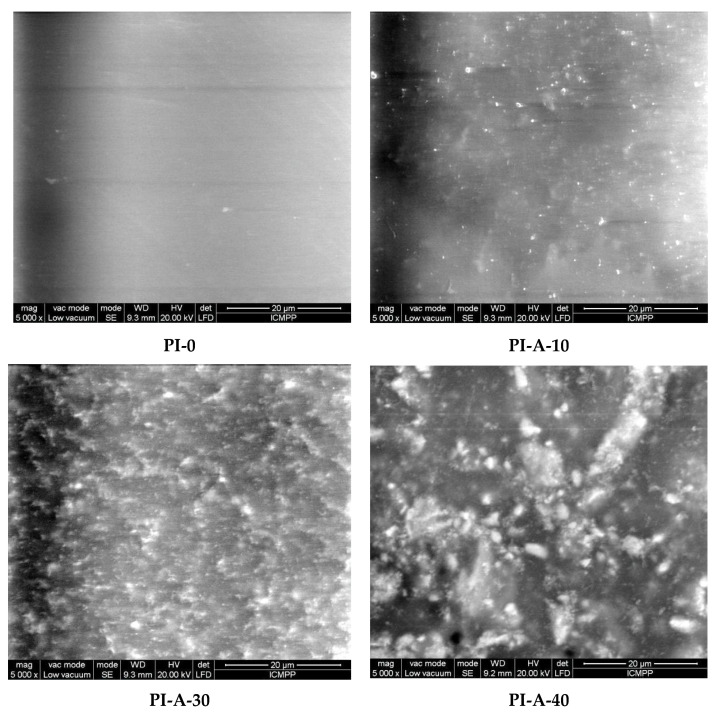
SEM micrographs of PI-0, PI-A-10, PI-A-30, and PI-A-40.

The morphology of the samples was investigated by SEM analysis, micrographs of PI-0, PI-A-10, PI-A-30, and PI-A-40 being illustrated in Figure 2. PI-0 had a homogeneous microstructure, while the surfaces of the composites were significantly rougher in comparison with the polyimide matrix PI-0, since the motion of the polymer chains was restricted by the inorganic filler. Some particle aggregations were observed, even in the case of PI-A-10. The dispersed particles had irregular shape and showed agglomerations attributed to the magnetic dipole–dipole interactions between inorganic particles. The semi-quantitative EDX analysis of PI-A-30 confirmed the presence of metal atoms Ce, Fe, Ni, and Cu. A mapping technique was used in order to illustrate the atom distribution on the composite surface. The EDX mapping of Ce, Fe, Ni, and Cu atoms showed a relatively uniform distribution of the particles (Figure S2 from ESD).

### 3.2. X-ray Analysis

The XRD patterns of the ferrite particles A, B, C, and D are depicted in Figure S3 from ESD. All ferrite samples used for those currently studied polyimide composites were previously reported [20,21]. Sample A, nominally Ni_0.7_Cu_0.3_Fe_1.92_Ce_0.08_O_4_ contains a 0.5 wt% of secondary cubic-CeO_2_ identified with ICSD card no. 01-073-6318. B sample (NiFe_1.92_Ce_0.08_O_4_) is considered 99.9% pure and C ferrite sample (Ni_0.4_Cu_0.6_Fe_1.92_Ce_0.08_O_4_) contains 1.6 wt% monoclinic CuO identified as ICSD card no. 01-070-6828. The XRD analysis and Rietveld refinement for D ferrite (CuFe_1.92_Ce_0.08_O_4_) confirmed the formation of cubic spinel ferrite with secondary 4.71 wt% tetragonal ferrite indexed as ICSD card no. 01-072-1174, 10.82 wt% monoclinic copper oxide, and 0.52 wt% cubic cerium oxide.

**Figure 3:**
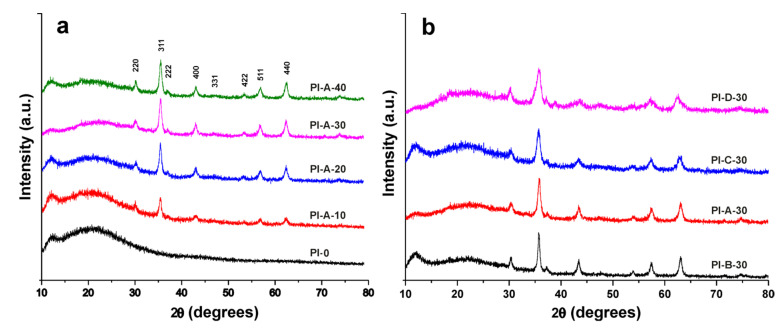
XRD patterns of PI-0, PI-A-10, PI-A-20, PI-A-30, and PI-A-40 (**a**) and PI-A-30, PI-B-30, PI-C-30, and PI-D-30 (**b**).

X-ray results of PI-0 and polyimide composites are illustrated in Figure 3. The diffractogram of PI-0 indicates an amorphous phase with two wide Bragg reflections, one around 12° and the second one around 21° (Figure 3a). The diffractograms of composites based on A-type ferrite (PI-A-10, PI-A-20, PI-A-30, and PI-A-40) have specific reflections corresponding to the cubic structure of Ni_0.7_Cu_0.3_Fe_1.92_Ce_0.08_O_4_ (A) particles. A noticeable increase in reflection intensity can be observed with increasing ferrite filling into the polyimide composite due to component ratio modification. At the same time, when compared to pure PI-0, the broad reflections of polyimide decreased in intensity with increasing magnetic particle content. This is due to a decrease in polymer content, coupled with the reduction in amorphization due to particle presence.

The diffractograms of composites with 30% ferrite (Figure 3b), PI-A-30, PI-B-30, PI-C-30, and PI-D-30, showed specific reflections corresponding to inorganic phases A, B, C, and D, respectively. The crystallinity of particles in composites was similar to that of particles in bulk form, suggesting that the particles are not modified by the thermal treatment during sample preparation.

### 3.3. Thermal and Magnetic Properties

Thermogravimetric analysis (TGA) was used to evaluate the thermal stability of the polyimide composites (Table 3). Figure S4 from ESD shows TG and DTG curves of PI-0, PI-A-10, PI-A-20, and PI-A-40. All samples exhibited high thermal stability as the initial decomposition temperature was found in 495–509 °C range. The temperature corresponding to 10% weight loss, *T*_10_, was 508–523 °C, while the temperature corresponding to maximum polymer decomposition, *T_max_*, was 509–527 °C. The char yield at 900 °C ranged between 42% and 53%, except PI-A-10 containing 10% particles, which showed a char yield of 26%, suggesting that the particles had a higher catalytic effect on the thermal decomposition reactions. A slight decrease in *T*_5_ and *T*_10_ values with increasing particle content could also be observed.

The glass transition temperature (*T_g_*) of the samples varied from 233 to 241 °C and slightly decreased by the incorporation of particles in the polymer matrix, suggesting that the presence of particles reduced the interactions between the macromolecular chains. Thus, *T_g_* of PI-0 was 241 °C, while *T_g_* of the composites varied in the 233–235 °C range (Table 3). However, the increase in particles content or the introduction of various type of particles have not had influence on *T_g_* values.

**Figure 4:**
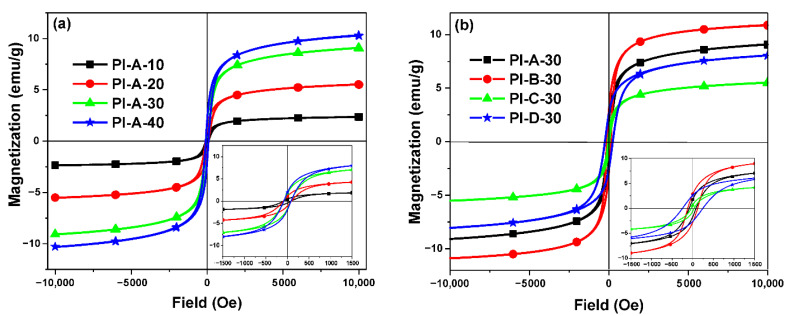
Hysteresis curves of PI-A-10, PI-A-20, PI-A-30, and PI-A-40, ±10 kOe representation—main figure, ±1.5 kOe—bottom right plot (**a**) and of PI-A-30, PI-B-30, PI-C-30, and PI-D-30, ±10 kOe representation—main figure, ±1.5 kOe—bottom right plot (**b**).

All composites exhibited a ferrimagnetic behavior due to particle interaction. The characteristic magnetic parameters including *M_s_*, *M_r_*, and *H_c_*, determined from the hysteresis loops, ranged in the intervals 2.37–10.90 emu g^−1^, 0.45–2.84 emu g^−1^, and 32–244 Oe, respectively (Table 3). The hysteresis loops of the samples containing 10%, 20%, 30%, and 40% A-type filler, PI-A-10, PI-A-20, PI-A-30, and PI-A-40, are shown in Figure 4a, and those corresponding to the samples with different types of particles at 30% concentration, PI-A-30, PI-B-30, PI-C-30, and PI-D-30, are illustrated in Figure 4b. As can be seen from Figure 4a and Table 3, increasing ferrite insertion ratio led to the progressive modification of *M_s_* and *M_r_*, while coercivity stayed in the same range (79–90 Oe). *M_s_* and *M_r_* increased in a linear fashion for 10–30% filler concentration, as was expected, since an increase in particle concentration leads to an increase in the magnetic moment per unit volume of the sample, and hence its magnetization increases [4]. Between insertion ratio of 30% and 40%, *M_s_* and *M_r_* exhibited a lower increase, indicating a tendency of the composites to reach magnetic saturation by canceling the magnetic moments due to interparticle interactions. This effect limited the efficiency of ratio modification up to 30% particles. It was also evident that the *M_s_* values of composites were lower than those of bulk magnetic particles.

The composites’ coercivity depended on particle type and exhibited lower variation with particle content. The magnetic values of composites suggest a different magnetic behavior induced by nickel substitution. For samples A, B, and C, the magnetic value changes are proportionally dropping, indicating that the sample magnetization and coercivity may decrease as a consequence of reduced interactions between Fe^Td^-Ni^Oh^, which are substituted with weaker Fe^Td^-Cu^Oh^ interactions, as suggested by Patange et al. [36].

### 3.4. Dielectric Constant and Dielectric Loss

**Figure 5:**
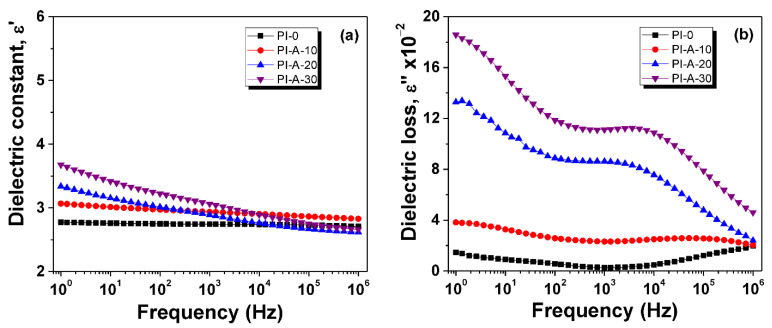
Dielectric constant (**a**) and dielectric loss (**b**) evolution with alternating frequency for PI-0, PI-A-10, PI-A-20, and PI-A-30, at 25 °C.

Broadband dielectric spectroscopy was employed for determining the behavior of electrical properties, namely, dielectric constant (*ε*′) and dissipation factor, also known as dielectric loss (*ε*″). The evolutions of dielectric constant and dielectric loss with frequency at 25 °C are represented in Figure 5 for the samples for which the ferrite loading varied from 0% to 30%. The dielectric constant decreased with increasing frequency (Figure 5a) since the molecular dipoles need a longer time than the alternating period to orient in the direction of the external electric field. As shown in Figure 5a, the isothermal plots displayed two different regions: a low-frequency region and a high-frequency region. In the low-frequency region, PI-0 and PI-A-10 exhibited a slight reduction in *ε*′, suggesting low dipolar activity. On the other hand, samples with superior ferrite content exhibited a higher value for *ε*′ and consequently, an elevated polarization ability. In the high-frequency region, the magnitude of *ε*′ was not significantly affected by the incorporation of ferrite in the PI backbone. Supplementary, the *ε*′(*f*) dependences of PI-A-30, PI-B-30, PI-C-30, and PI-D-30 are presented in Figure S5a from ESD. The magnitude of *ε*′ is diminished with the change of substitution ratio from x = 0 to 0.6, especially in the low-frequency region.

According to Figure 5b and Figure S5b from ESD, the magnitude of dielectric loss was considerably affected by the ferrite content and the nickel substitution ratio, respectively. At the same time, the *ε*″(*f*) trends showed specific dielectric signals for secondary *γ*- and *β*-relaxations. Therefore, at high frequencies, *γ*-relaxation appeared as a well-defined dielectric peak while the head-part of *β*-relaxation raised at low frequencies.

The values of dielectric parameters of the composites of different ferrite content are presented in Table 4. The lowest values of *ε*′ were observed for PI-0 throughout the considered frequency range (e.g., for *f* = 1 Hz, *ε*′ = 3), which were close to other reported polyimides [37,38]. Addition of ferrite particles in the polymer matrix increased the dielectric constant. Moreover, the values of the dielectric constant were influenced by the type of magnetic ferrite, especially due to nickel substitution (Table 4). At room temperature, *ε*′ of composites with various ferrite loadings ranged from 3.3 to 4.3, accompanied by low dielectric losses, between 10^−1^ and 10^−2^, and low conductivities, around 10^−13^–10^−14^ S cm^−1^ (1 Hz). From these data, it is clear that ferrite particle embedding in the polyimide matrix induces dielectric constant and conductivity enrichment, the greatest enhancement being observed at the highest ferrite concentration.

**Figure 6:**
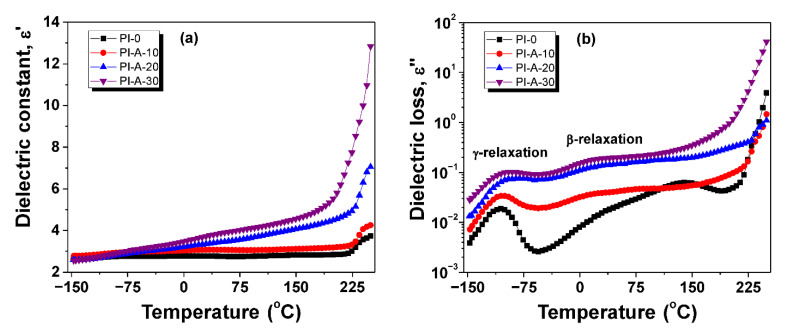
Dielectric constant (**a**) and dielectric loss (**b**) evolution with temperature for PI-0, PI-A-10, PI-A-20, and PI-A-30, at a frequency of 1 Hz.

Figure 6 depicts the temperature behavior of dielectric constant and loss factor of the samples at a frequency of 1 Hz. First of all, it can be observed that from −150 to 200 °C, *ε*′ increased slowly with temperature, while the loss factor values were below 0.1, allowing the use of this type of composites as stable dielectrics in a broad range of temperature. Second, the magnitude of temperature dependences varied substantially with the ferrite content. Furthermore, the isochronal plots of dielectric loss revealed the presence of some important dipolar relaxation processes. At low temperatures, around −100 °C, the secondary *γ*-relaxation appeared as a narrow dielectric peak, which is generally activated by small groups of macromolecules that have attached dipolar water molecules from atmosphere. As temperature increased, *β*-relaxation process became visible (e.g., for PI-A-10, *β*-relaxation appeared around 30 °C) as a broad band with reduced intensity. According to already published literature, the *β*-relaxation is assigned to the cooperative rotational motions involving segmental motions from the main chain [39]. At higher temperatures, the dielectric loss increased considerably due to raised mobility of charge carriers.

**Figure 7:**
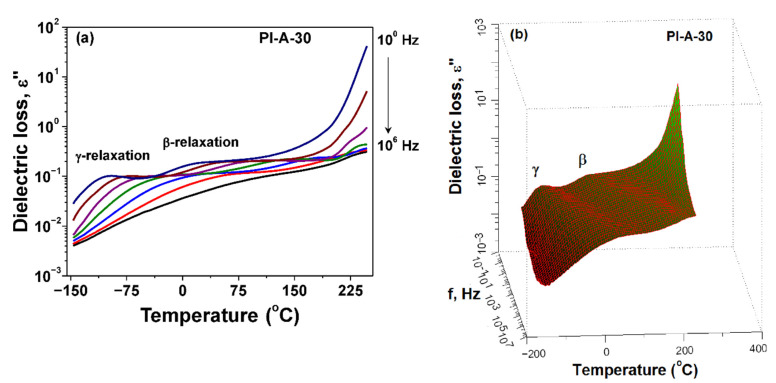
Dielectric loss evolution with temperature at selected frequencies (**a**) and 3D presentation of *ε*″ as function of frequency and temperature (**b**) for PI-A-30.

Figure 7 depicts the evolution of dielectric loss with temperature at selected frequencies for the sample PI-A-30. The secondary *γ*- and *β*-relaxations were clearly recognized as intensive dielectric peaks that shifted gradually to higher temperatures as frequency increased from 1 to 10^6^ Hz. The dipolar processes corresponding to PI-A-30 were easier visualized on the 3D version of dielectric loss as function of both the applied frequency and the temperature (Figure 7b).

### 3.5. Activation Energy

Detailed *ε*″(*f*) dependencies recorded at various temperatures are shown for PI-A-10 in Figure S6 from ESD. The secondary *γ*-relaxation appeared as one intense peak at −80 °C that shifted to higher frequencies and lowered in intensity as temperature increased (Figure S6a from ESD). The *β*-relaxation appeared as a broad peak at low frequencies (e.g., at 80 °C, the peak maximum appeared at around 10^2^ Hz) and its signal was strongly affected by the conductivity signal. The remaining composites with various filling ratios provided analogous dipolar signals.

As previously discussed, the dipolar relaxation peaks are more or less overlapped with the conductivity signal. As a consequence, the *ε*″(*f*) dependencies were fitted considering the Havriliak–Negami (HN) expression:

(1) $$\varepsilon^{*}=\varepsilon^{\prime }-i\varepsilon^{\prime \prime }=\varepsilon_{u}+\frac{\varepsilon_{r}-\varepsilon_{u}}{{\left[1+{\left(i\omega \tau_{HN}\right)}^{a}\right]}^{b}},$$

where *ε_r_* and *ε_u_* are the relaxed and unrelaxed values of the dielectric constant, *ω* represents the angular frequency, *τ_HN_* is the HN relaxation time of the process, while *a* and *b* represent the broadening and skewing parameters, respectively [40]. Deconvolution was performed with the WinFit software provided by Novocontrol and the relaxation time of each dielectric peak maxima, *τ_max_*, was calculated with the following relation:

(2) $$\tau_{max}=\tau_{HN}{\left[\frac{sin\frac{\pi ab}{2+2b}}{sin\frac{\pi a}{2+2b}}\right]}^{\frac{1}{a}}.$$

Figure S7 from ESD depicts an example of HN fitting for the secondary relaxation processes. The HN fit from Figure S8 from ESD encloses a HN term attributed to *γ*-relaxation and a supplementary term for both the *β*-relaxation and conductivity. The dependency of *ε*″(*f*) on *β*-relaxation was further processed with two HN functions (one for *β*-relaxation at around 10^2^ Hz and one for *γ*-relaxation limited at high frequencies), as well as an additional function for conductivity.

**Figure 8:**
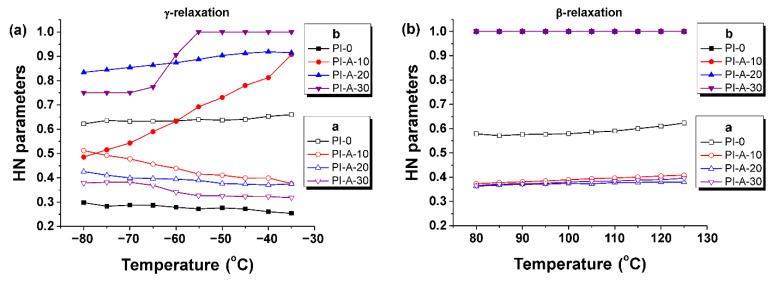
Broadening and skewing parameters as function of temperature for *γ* (**a**) and *β* (**b**) relaxations of PI-0, PI-A-10, PI-A-20, and PI-A-30.

The numerical values of broadening and skewing parameters from Equation (1) are represented as a function of temperature in Figure 8. The skewing parameter of PI-0 was around 0.3, indicating an asymmetry of the *γ*-relaxation. This parameter increased in value with the addition of ferrite, as well as with rising temperature, suggesting an improved symmetry. In addition, for composites with 30% ferrite loadings and different types of magnetic ferrite, the symmetry of the relaxation peaks increased considerably (see the Figure S8 from ESD). On the other hand, the width parameter was substantially reduced with increasing loadings of ferrite, suggesting complex motions of the side chain groups. Regarding the HN fitting process of *β*-relaxation, the dipolar peaks were symmetrical and, as consequence, the dielectric spectra were processed with *b* = 1 in the entire temperature range.

**Figure 9:**
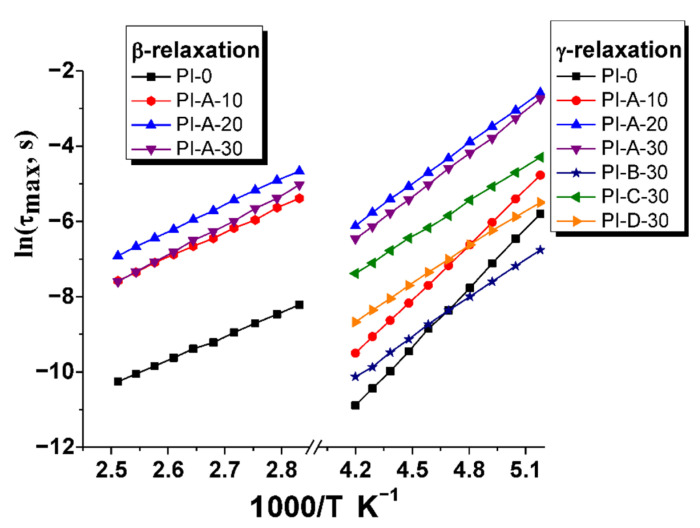
Arrhenius plots of *γ-* and *β-* relaxations.

The Arrhenius equation was used to determine the activation energy, *E_a_*, of the relaxation processes:

(3) $$\tau_{max}\left(T\right)=\tau_{0}\exp\left(\frac{E_{a}}{RT}\right),$$

where *τ_max_* was previously obtained from Equation (2), *τ*_0_ is a pre-exponential factor and *R* is the gas constant. The relaxation times associated with the peak maxima are plotted as a function of inverse temperature in Figure 9, and the numerical values of Equation (3) can be consulted in Table 5. The secondary *γ*-relaxation is characterized by small activation energy values, between 27 and 43 kJ mol^−1^, revealing that this relaxation is non-cooperative and can only be stimulated by limited fluctuations of small groups. The activation energy values were lower for composites than that of neat polyimide, indicating that the inorganic particles led to fewer restrictions to this dipolar relaxation. On the other hand, the values of *E_a_* for *β*-relaxation were higher than that for *γ*-relaxation, confirming the existence of cooperative rotational motions. The proportional increase in *E_a_* with the concentration of magnetic ferrite indicates the reinforcement of the polymer chains by the nanoparticles, and as a consequence, the hindered movement of chemical dipoles. It is important to note that *β*-relaxation could not be accurately determined for composites with different types of magnetic ferrite due to the signal overlap with conductivity.

### 3.6. Conductivity

**Figure 10:**
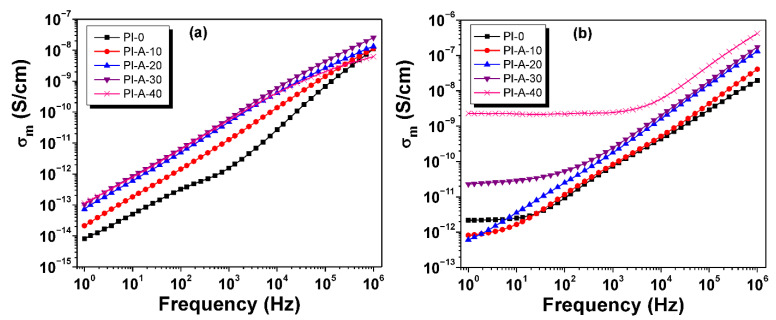
The frequency evolution of measured conductivity at 25 °C (**a**) and 250 °C (**b**) for PI-0, PI-A-10, PI-A-20, PI-A-30, and PI-A-40.

The evolution of measured conductivity as a function of alternating field frequency at a moderate temperature (25 °C) and at a high temperature (250 °C) is presented in Figure 10. At 25 °C, the measured conductivity increased almost linearly with frequency for all considered samples (Figure 10a). The conductivity values were low (e.g., for PI-A-30, at f = 1 Hz, the measured conductivity was ∼10^−13^ S cm^−1^), due to the high insulator-type characteristic of the polyimide matrix (Table 4). At high temperatures (Figure 10b), the conductivity substantially increased, especially for samples containing a high amount of ferrite. It is clear that the addition of ferrite progressively increased the conductivity of the samples. The increase in conductivity with increasing temperature is a normal effect for polymer-based composites. For polyimides, it is known that the cooperative segment motions of the polymer backbone are activated at temperatures above glass transition, and consequently, the polymer matrix favors the movements of charge carriers. On the other hand, the incorporation of ferrite furnishes a proportional concentration of Fe^3+^ ions into the polyimide matrix. Thus, a higher amount of ferrite leads to the increased conductivity of PI-A-30 and PI-A-40.

## 4. Conclusions

Novel magnetic polyimide/copper-nickel ferrite composites were developed using cerium-doped ferrite particles, namely, NiFe_1.92_Ce_0.08_O_4_, Ni_0.7_Cu_0.3_Fe_1.92_Ce_0.08_O_4_, Ni_0.4_Cu_0.6_Fe_1.92_Ce_0.08_O_4_, and CuFe_1.92_Ce_0.08_O_4_, as inorganic fillers. An aromatic polyimide containing pendant carboxylic groups was used as polymer matrix in order to ensure a good dispersion of the filler particles. The influence of filler content on the composite properties was evidenced through specific ferrimagnetic behavior in the case of all the samples. *M_s_* and *H_c_* of composite materials strongly depended on ferrite composition and progressively increased up to a 40% filler ratio, allowing an efficient control over the polymer-ferrite composite magnetic values. The tendency to reach maximum *M_s_* and *H_c_* for the polymer-ferrite material can be explained by particle agglomeration at high ferrite content, as revealed by SEM investigations. According to TGA analysis, the composites showed high heat resistance, with initial decomposition temperatures in the range of 495–509 °C. The dielectric properties, as determined by broadband dielectric spectroscopy measurements, were related to ferrite structure and loading level. The observed low values for the dielectric parameters in a broad temperature range (between −150 and 200 °C) demonstrate a high thermal stability of these composites, allowing their use as stable dielectrics in a broad temperature range.

## Figures and Tables

**Table 1:** Magnetic values of Ni_1-x_Cu_x_Fe_1.92_Ce_0.08_O_4_ ferrite particles.

Ferrite Sample	Chemical Formula	*M_s_* (emu g^−1^)	*M_r_* (emu g^−1^)	*H_c_* (Oe)
A: [x = 0.3]	Ni_0.7_ Cu_0.3_ Fe_1.92_ Ce_0.08_ O_4_	25.4	3.34	53.45
B: [x = 0.0]	NiFe_1.92_ Ce_0.08_ O_4_	33.86	5.23	65.75
C: [x = 0.6]	Ni_0.4_Cu_0.6_ Fe_1.92_ Ce_0.08_ O_4_	20.77	1.73	32.32
D: [x = 1.0]	CuFe_1.92_ Ce_0.08_ O_4_	19.44	6.47	246.2

**Table 2:** Type and concentration of ferrite particles for each composite sample.

Polyimide Composite	Type of Ferrite Particles	Concentration of Particles in Polyimide Composite (wt%)
PI-0	–	–
PI-A-10	Ni_0.7_ Cu_0.3_ Fe_1.92_ Ce_0.08_ O_4_	10
PI-A-20	Ni_0.7_ Cu_0.3_ Fe_1.92_ Ce_0.08_ O_4_	20
PI-A-30	Ni_0.7_ Cu_0.3_ Fe_1.92_ Ce_0.08_ O_4_	30
PI-A-40	Ni_0.7_ Cu_0.3_ Fe_1.92_ Ce_0.08_ O_4_	40
PI-B-30	NiFe_1.92_ Ce_0.08_ O_4_	30
PI-C-30	Ni_0.4_Cu_0.6_ Fe_1.92_ Ce_0.08_ O_4_	30
PI-D-30	CuFe_1.92_ Ce_0.08_ O_4_	30

**Table 3:** The main thermal and magnetic characteristics of the samples.

Sample	*T*_5_^a^ (°C)	*T*_10_^b^ (°C)	*T_max_* ^c^ (°C)	Char Yield at 900 °C (%)	*T_g_*^d^ (°C)	*M_s_* (emu g^−1^)	*M_r_* (emu g^−1^)	*H_c_* (Oe)
PI-0	509	522	527	45	241	-	-	-
PI-A-10	507	523	522	26	234	2.37	0.45	84
PI-A-20	506	520	515	43	235	5.52	1.11	90
PI-A-30	501	513	509	53	234	9.05	1.76	79
PI-A-40	498	511	511	49	233	10.28	1.98	83
PI-B-30	500	512	512	42	235	10.90	2.84	119
PI-C-30	502	515	513	47	234	5.53	0.63	32
PI-D-30	495	508	510	48	233	8.02	2.61	244

^a^ Initial decomposition temperature = temperature of 5% weight loss; ^b^ temperature of 10% weight loss; ^c^ temperature of maximum polymer decomposition, ^d^ glass transition temperature (the inflection point).

**Table 4:** Dielectric constant, dielectric loss, and conductivity values, at 25 °C and selected frequencies.

Sample	Dielectric Constant		Dielectric Loss		Conductivity (S cm^−1^)	
	1 Hz	10^4^ Hz	1 Hz	10^4^ Hz	1 Hz	10^4^ Hz
PI-0	3.0	3.0	1.6 × 10^−2^	0.5 × 10^−2^	8.8 × 10^−15^	2.9 × 10^−11^
PI-A-10	3.3	3.1	4.1 × 10^−2^	2.7 × 10^−2^	2.3 × 10^−14^	1.5 × 10^−10^
PI-A-20	3.5	2.9	13.9 × 10^−2^	7.9 × 10^−2^	7.8 × 10^−14^	4.4 × 10^−10^
PI-A-30	3.9	3.1	19.6 × 10^−2^	11.5 × 10^−2^	1.1 × 10^−13^	6.4 × 10^−10^
PI-B-30	4.3	3.6	19.7 × 10^−2^	13.3 × 10^−2^	1.1 × 10^−13^	7.4 × 10^−10^
PI-C-30	3.4	3.1	2.7 × 10^−2^	10.8 × 10^−2^	1.5 × 10^−14^	6.0 × 10^−10^
PI-D-30	3.7	3.1	18.2 × 10^−2^	14.8 × 10^−2^	1.0 × 10^−13^	8.2 × 10^−10^

**Table 5:** The numerical values of Equation (3) for the samples.

Sample	*γ*-Relaxation		*β*-Relaxation	
	*τ*_0_ (s)	*Eγ* (kJ mol^−1^)	*τ*_0_ (s)	*E_β_* (kJ mol^−1^)
PI-0	6.0 × 10^−15^	43.3	3.9 × 10^−12^	53.1
PI-A-10	1.1 × 10^−13^	40.3	1.4 × 10^−11^	57.7
PI-A-20	6.1 × 10^−10^	30.0	1.7 × 10^−11^	59.2
PI-A-30	1.9 × 10^−10^	31.6	1.1 × 10^−12^	66.3
PI-B-30	1.8 × 10^−11^	29.0	–	–
PI-C-30	8.7 × 10^−10^	27.0	–	–
PI-D-30	1.9 × 10^−10^	27.3	–	–

## Data Availability

The data presented in this study are available on request from the corresponding author.

## Supplementary Materials

The following are available online at https://www.mdpi.com/article/10.3390/polym13101646/s1, Figure S1: FTIR spectra of PI-0 and PI-A-30., Figure S2: EDX diagram (a) and EDX mapping (Ce, Fe, Ni and Cu atoms) (b) of PI-A-30., Figure S3: XRD patterns of inorganic fillers Ni_1-x_Cu_x_Fe_1.92_Ce_0.08_O_4_ (with x = 0.0, 0.3, 0.6, 1.0), A, B, C and D., Figure S4: TG (a) and DTG (b) curves of PI-0, PI-A-10, PI-A-20 and PI-A-40., Figure S5: Dielectric constant (a) and dielectric loss (b) evolution with alternating frequency for samples with 30% ferrite content at 25°C., Figure S6: Representative ε”(f) dependences for isothermal γ-relaxation (a) and β-relaxation (b) of PI-A-10., Figure S7: Representative deconvolution process for γ (a) and β (b) relaxations of PI-A-10., Figure S8: The evolution of broadening and skewing parameters as function of temperature for γ-relaxation of composites with different types of magnetic ferrite.
