# Supplementary material for: Novel Polyimide/Copper-Nickel Ferrite Composites with Tunable Magnetic and Dielectric Properties
Source: Polymers (Basel). 2021 May 19;13(10):1646. doi: 10.3390/polym13101646 (PMC8158717; doi:10.3390/polym13101646)
Supplement: Supplementary file 1 [file polymers-13-01646-s001.zip › polymers-1210161-supplementary.pdf]

Electronic Supplementary Data

Novel polyimide/copper-nickel ferrite composites with tunable magnetic and dielectric properties

Corneliu Hamciuc[a], Mihai Asandulesa[a], Elena Hamciuc[a], Aurel Pui[b*], Tiberiu Roman[b,c], Marius Andrei Olariu[d,e]

[a] *"Petru Poni" Institute of Macromolecular Chemistry, 41A Aleea Gr. Ghica Voda, 700487 Iasi, Romania*
[b] *"Al. I. Cuza" University of Iasi, Faculty of Chemistry, 11 Bd. Carol I, 700506 Iasi, Romania*
[c] *"Al. I. Cuza" University of Iasi, Integrated Centre of Environmental Science Studies in the North-Eastern Region - CERNESIM, 11 Bd. Carol I, 700506 Iasi, Romania*
[d] *Prosupport Consulting SRL, 29 Peter Culianu Street, 707410 Valea Lupului - Iasi, Romania*
[e] *Electrical Engineering Faculty, "Gh. Asachi" Technical University, B-Dul D. Mangeron 67, Iasi 700050, Romania*

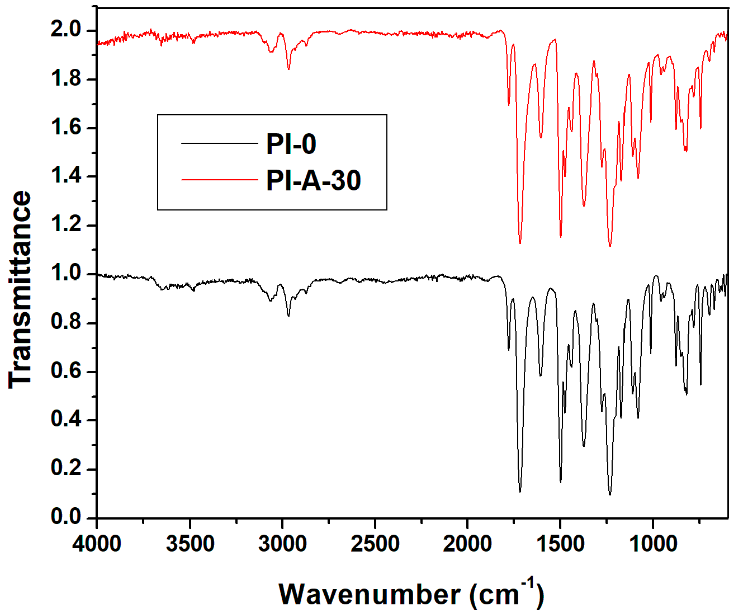

Fig. S1. FTIR spectra of PI-0 and PI-A-30.

(a) (b)

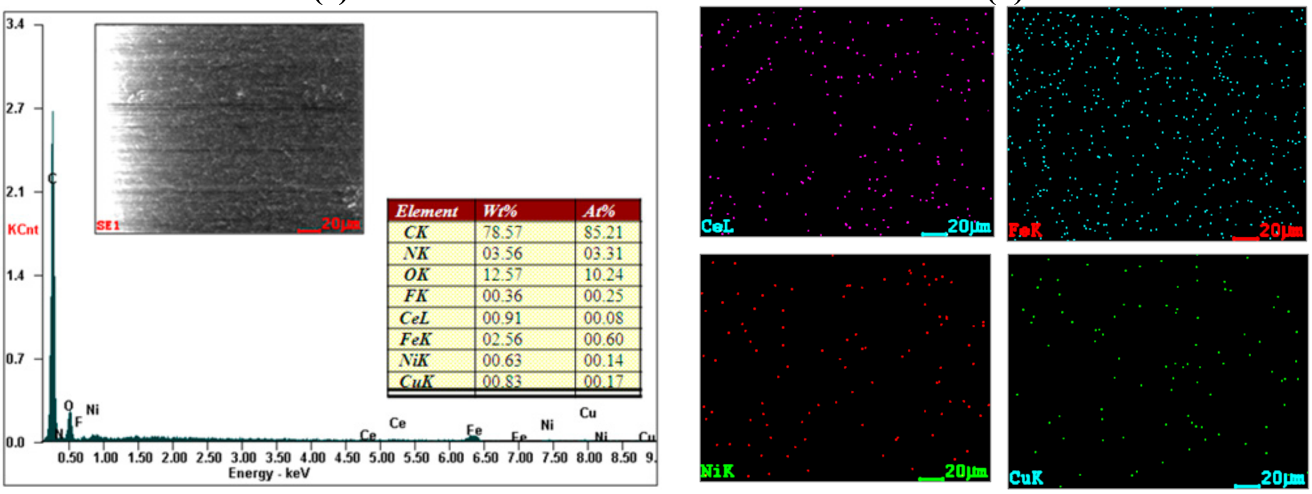

Element	Wt%	At%
CK	78.57	85.21
NK	03.56	03.31
OK	12.57	10.24
FK	00.36	00.25
CeL	00.91	00.08
FeK	02.56	00.60
NiK	00.63	00.14
CuK	00.83	00.17

Fig. S2. EDX diagram (a) and EDX mapping (Ce, Fe, Ni and Cu atoms) (b) of PI-A-30.

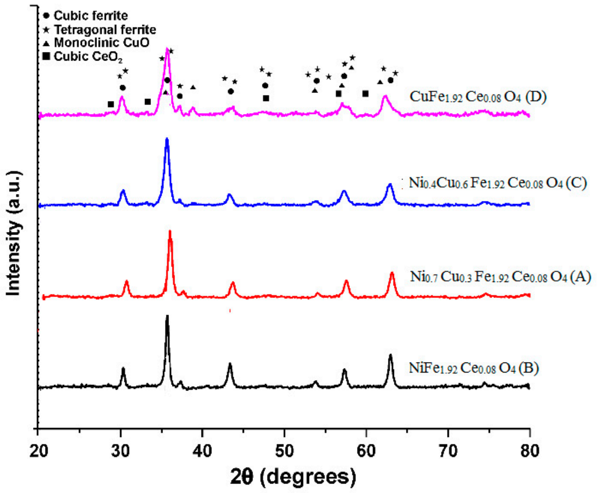

Fig. S3. XRD patterns of inorganic fillers $Ni_{1-x}Cu_xFe_{1.92}Ce_{0.08}O_4$ (with x = 0.0, 0.3, 0.6, 1.0), A, B, C and D.

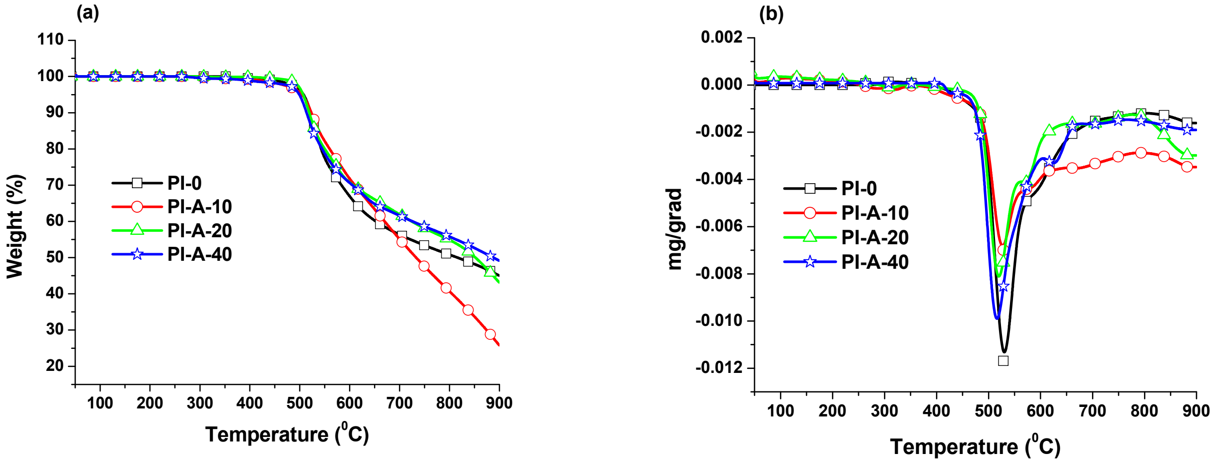

Fig. S4. TG (a) and DTG (b) curves of PI-0, PI-A-10, PI-A-20 and PI-A-40.

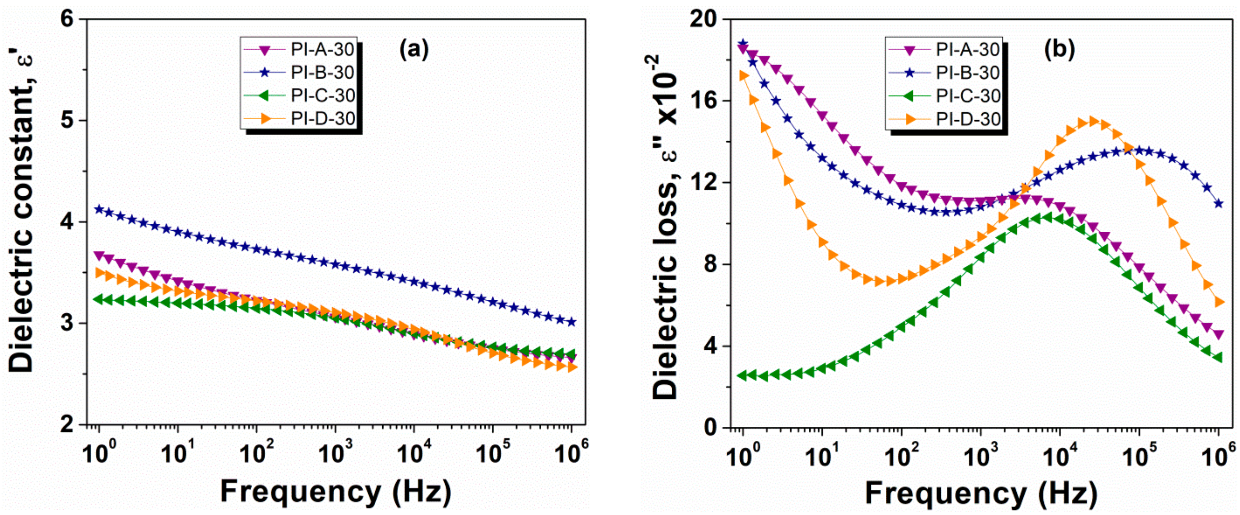

Fig. S5. Dielectric constant (a) and dielectric loss (b) evolution with alternating frequency for samples with 30% ferrite content at 25°C.

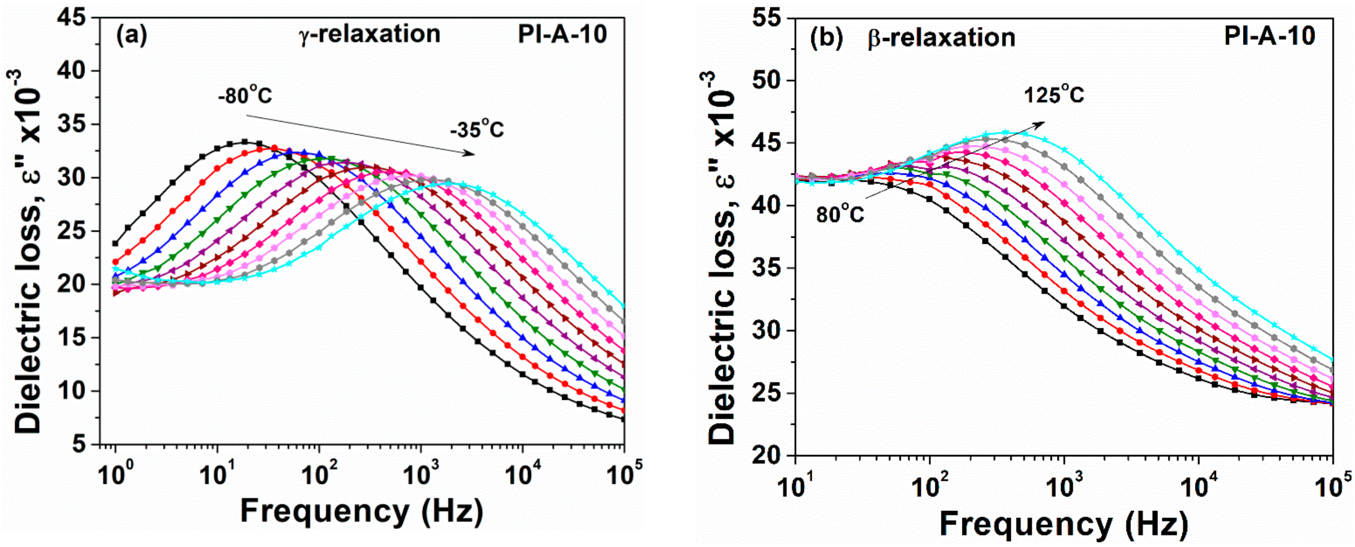

Fig. S6. Representative *ε″*(*f*) dependences for isothermal *γ*-relaxation (a) and *β*-relaxation (b) of PI-A-10.

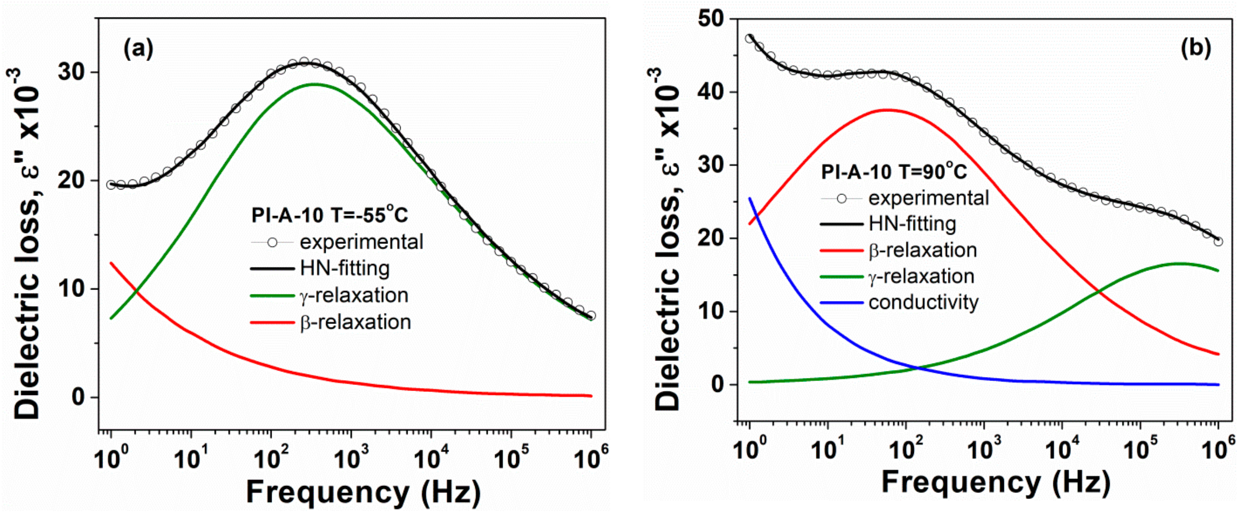

Fig. S7. Representative deconvolution process for *γ* (a) and *β* (b) relaxations of PI-A-10.

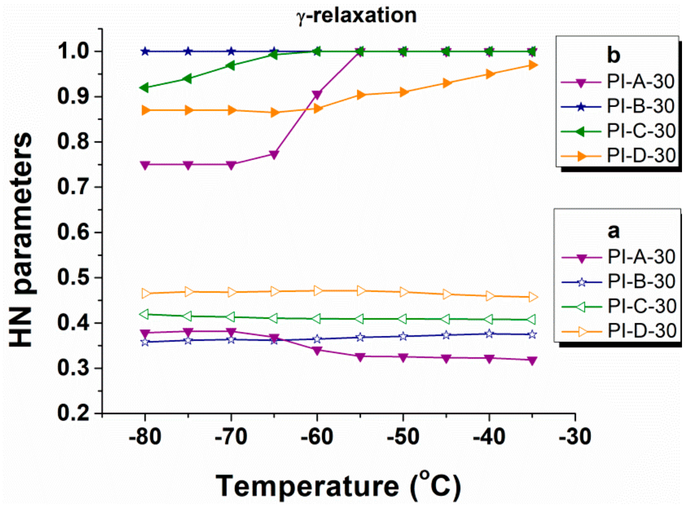

Fig. S8. The evolution of broadening and skewing parameters as function of temperature for *γ*-relaxation of composites with different types of magnetic ferrite.
